# Seeding Propensity and Characteristics of Pathogenic αSyn Assemblies in Formalin-Fixed Human Tissue from the Enteric Nervous System, Olfactory Bulb, and Brainstem in Cases Staged for Parkinson’s Disease

**DOI:** 10.3390/cells10010139

**Published:** 2021-01-12

**Authors:** Alexis Fenyi, Charles Duyckaerts, Luc Bousset, Heiko Braak, Kelly Del Tredici, Ronald Melki, on behalf of the Brainbank Neuro-CEB Neuropathology Network

**Affiliations:** 1 Institute Francois Jacob (MIRCen), CEA and Laboratory of Neurodegenerative Diseases, CNRS, 92265 Fontenay-Aux-Roses CEDEX, France; Alexis.FENYI@cnrs.fr (A.F.); Luc.BOUSSET@cnrs.fr (L.B.); 2 Laboratoire de Neuropathologie Escourolle, Hôpital de la Salpêtrière, AP-HP, & Alzheimer Prion Team, ICM, 47 Bd de l’Hôpital, 75651 CEDEX 13 Paris, France; charles.duyckaerts@aphp.fr; 3 Clinical Neuroanatomy Section/Department of Neurology, Center for Biomedical Research, University of Ulm, Helmholtzstraße 8/1, 89081 Ulm, Germany; heiko.braak@uni-ulm.de (H.B.); kelly.del-tredici@uni-ulm.de (K.D.T.)

**Keywords:** alpha-synuclein, enteric nervous system, incidental Lewy body disease, Lewy body disease, Parkinson’s disease, protein misfolding cyclic amplification (PMCA), prion-like, synucleinopathy, synuclein strains

## Abstract

We investigated α-synuclein’s (αSyn) seeding activity in tissue from the brain and enteric nervous system. Specifically, we assessed the seeding propensity of pathogenic αSyn in formalin-fixed tissue from the gastric cardia and five brain regions of 29 individuals (12 Parkinson’s disease, 8 incidental Lewy body disease, 9 controls) using a protein misfolding cyclic amplification assay. The structural characteristics of the resultant αSyn assemblies were determined by limited proteolysis and transmission electron microscopy. We show that fixed tissue from Parkinson’s disease (PD) and incidental Lewy body disease (ILBD) seeds the aggregation of monomeric αSyn into fibrillar assemblies. Significant variations in the characteristics of fibrillar assemblies derived from different regions even within the same individual were observed. This finding suggests that fixation stabilizes seeds with an otherwise limited seeding propensity, that yield assemblies with different intrinsic structures (i.e., strains). The lag phase preceding fibril assembly for patients ≥80 was significantly shorter than in other age groups, suggesting the existence of increased numbers of seeds or a higher seeding potential of pathogenic αSyn with time. Seeding activity did not diminish in late-stage disease. No statistically significant difference in the seeding efficiency of specific regions was found, nor was there a relationship between seeding efficiency and the load of pathogenic αSyn in a particular region at a given neuropathological stage.

## 1. Introduction

The aggregation of the protein α-synuclein (αSyn) is closely associated with a variety of age-related neurodegenerative disorders known as synucleinopathies. Intraneuronal inclusions in cell bodies and in neurites characterize Parkinson’s disease (PD), including incidental Lewy body disease (ILBD), whereas inclusions in oligodendrocytes are typical of multiple system atrophy (MSA).

Two sets of evidence suggest that Lewy pathology spreads within the nervous system. The first comes from immunohistochemical studies showing that Lewy pathology is not only present in widespread brain areas in synucleinopathies but also in peripheral nerve cells and cellular processes of the autonomic nervous system. Inasmuch as the affected brain regions are interconnected by neural pathways, it was proposed that a causative agent, possibly possessing “prion-like properties,” initially affecting the olfactory bulb and autonomic nerve cells innervating the upper gastrointestinal tract might spread progressively within connected regions [1,2,3]. Additional supporting evidence for the spread of pathology comes from the presence of Lewy bodies in fetal neurons grafted in the brain of patients developing synucleinopathies over 10 years prior to death [4,5,6]. αSyn-rich aggregates appeared to have moved from affected cells within the host brain to the naïve grafted neurons, where they then triggered the aggregation of endogenous αSyn.

The evidence for the transfer and spread of pathology led to the design of numerous cell culture and in vivo studies supporting the view that αSyn fibrils produced in test tubes and αSyn-rich deposits purified from model animals or patients have prion-like properties, such as propagation [7,8,9,10,11,12,13,14,15,16,17,18,19,20,21,22,23,24,25,26,27,28,29,30].

The occurrence of intracellular deposits containing αSyn at the post-mortem histopathological assessment of formalin-fixed tissues from the central nervous system allows for the definite diagnosis of PD and ILBD. We and others recently demonstrated that, using either protein misfolding cyclic amplification (PMCA) or real-time quaking-induced conversion (RT-QuIC) assays, αSyn deposits are found in the brains or gastrointestinal tracts of patients suffering from PD, dementia with Lewy bodies (DLB), or MSA seed monomeric human αSyn in vitro [31,32,33,34,35].

To determine to what extent the seeding propensity and intrinsic structural characteristics of aggregated αSyn are preserved after fixation, we compared six immunohistopathologically characterized tissues taken from the brain (CNS) and enteric nervous system (ENS) of controls and individuals with PD or ILBD for the seeded aggregation of monomeric αSyn. We also assessed the structural characteristics of the resulting assemblies using limited proteolysis and transmission electron microscopy (TEM). Here, we could show that fixed tissues from patients who displayed Lewy pathology seed the aggregation of monomeric αSyn into fibrillar assemblies. PMCA-amplified fibrillar αSyn assemblies from fixed tissue originating from ILBD and PD cases did not exhibit shapes or proteolytic profiles that could be considered characteristic or distinctive of each group. This contrasts with what we have observed previously upon amplification of pathogenic αSyn deposits from frozen brain tissues [31]. Furthermore, the limited proteolytic patterns of the fibrillar αSyn assemblies derived from the CNS and ENS of different ILBD and PD patients varied significantly. This may indicate that fixation unveils the existence of distinct strains within different tissues originating from a given patient. To our knowledge, this study is the first to analyze αSyn seeding in tissues from both the CNS and the ENS in such cases.

## 2. Materials and Methods

### 2.1. Human Autopsy Samples

Autopsy tissue from the brain and stomach used for this study was obtained from *n* = 12 individuals with sporadic PD (7 females, 5 males, age range 60–84 years), *n* = 8 with ILBD (2 females, 6 males, age range 44–82 years), and *n* = 9 controls (5 females, 4 males, age range 44–67 years) in compliance with ethics committee guidelines at the Universities of Ulm and Paris, as well as with German federal and state law governing human tissue usage. Informed written consent for autopsy was previously obtained from patients or from their next of kin. The postmortem interval (PMI), defined as the time between death and autopsy, ranged from 4 to 96 h (median: 19 h for controls, 26 for ILBD, and 30 h for PD). Demographic and clinico-neuropathological data for all 29 cases are provided in Table 1.

### 2.2. Tissue Fixation, Embedding, and Sectioning

Brains and stomachs were fixed in a 4% buffered aqueous solution of formaldehyde for 14 days. Tissue blocks excised from the brain were embedded in polyethylene glycol (PEG 1000, Merck, Carl Roth Ltd., Karlsruhe, Germany) and sectioned at 100 µm on a tetrander (Jung, Heidelberg, Germany), as described previously [36,37,40]. 5 mm × 30 mm tissue blocks cut tangentially were excised from the gastric cardia (the adventitia, muscle layers, submucosa, and Auerbach plexus were included) for cryosectioning at 100 µm thickness on a freezing microtome [39]. Brains, stomachs, and the remaining tissue sections were stored for subsequent use in a 4% aqueous solution of formaldehyde at 8–15 °C.

### 2.3. Immunohistochemistry and Neuropathological Staging

Neuropathological staging and disease classification were performed according to previously published protocols [2,38] using each of the following primary antibodies: (1) a monoclonal anti-syn-1 antibody (1:2000; Clone number 42; BD Biosciences, Eysins, Switzerland) for detection of Lewy body disease-related α-synuclein inclusions; (2) a monoclonal anti-PHF-Tau antibody, 1:2000; Clone AT8; Pierce Biotechnology [Thermo Scientific] Waltham, MA [41]) to visualize the broadest spectrum of intraneuronal pathological (misfolded) tau: neurofibrillary tangles (NFTs) of the Alzheimer type, neuropil threads (NTs) in dendritic processes, somatic aggregates, non-argyrophilic axonal aggregates and pretangles, argyrophilic grains; (3) a monoclonal anti-Aβ antibody 4G8 (1:5000; Clone 4G8; BioLegend, San Diego, CA, USA) for detection of Aβ deposition, as recommended previously [38]. All brain and stomach sections were stained free-floating. Care was taken to exclude MSA and the non-AD tauopathies Pick’s disease, progressive supranuclear palsy, corticobasal degeneration, and Niemann-Pick disease type C. Additional sets of 100 µm thick tissue sections from the brain were processed according to a modified Gallyas silver-iodide staining protocol [36,37] for recognition of phosphorylated somatic argyrophilic (fibrillar) NTs [42,43] and NFTs, as well as extraneuronal ghost tangles (“tombstone” tangles) of the Alzheimer type [36,38].

Free-floating tissue sections for immunohistochemistry (IHC) were treated for 30 min in a mixture of 10% methanol plus 10% concentrated (30%) H_2_O_2_ and 80% Tris. Following pretreatment with 100% formic acid for 3 min (syn-1, 4G8) to facilitate the immunoreactions, blocking with bovine serum albumin was performed to inhibit endogenous peroxidase and to prevent nonspecific binding. Subsequently, each set of free-floating sections was incubated for 18 h at 20 °C using the primary antibodies above. After processing with a corresponding secondary biotinylated antibody (anti-mouse or anti-rabbit IgG, 1:200; Linaris, Vector Laboratories, Dossenheim, Germany) for 1.5 h, all immunoreactions were visualized with the avidin-biotin complex (ABC, Vectastain, Vector Laboratories, Dossenheim, Germany) for 2 h and the chromogen 3,30-diaminobenzidine tetrahydrochloride ([DAB], D5637, Sigma, Taufkirchen, Germany). The omission of the primary antiserum resulted in non-staining. Positive and negative control sections were included for all immunoreactions. The selected brain sections were also counterstained with a basophilic Nissl stain (Darrow red, 211885, Sigma-Aldrich, Steinheim, Germany) for topographical overview and as a marker for neuronal loss [36]. Then, tissue sections were cleared, mounted, and cover-slipped (Histomount, National Diagnostics, Nottingham, UK).

**Figure 1:**
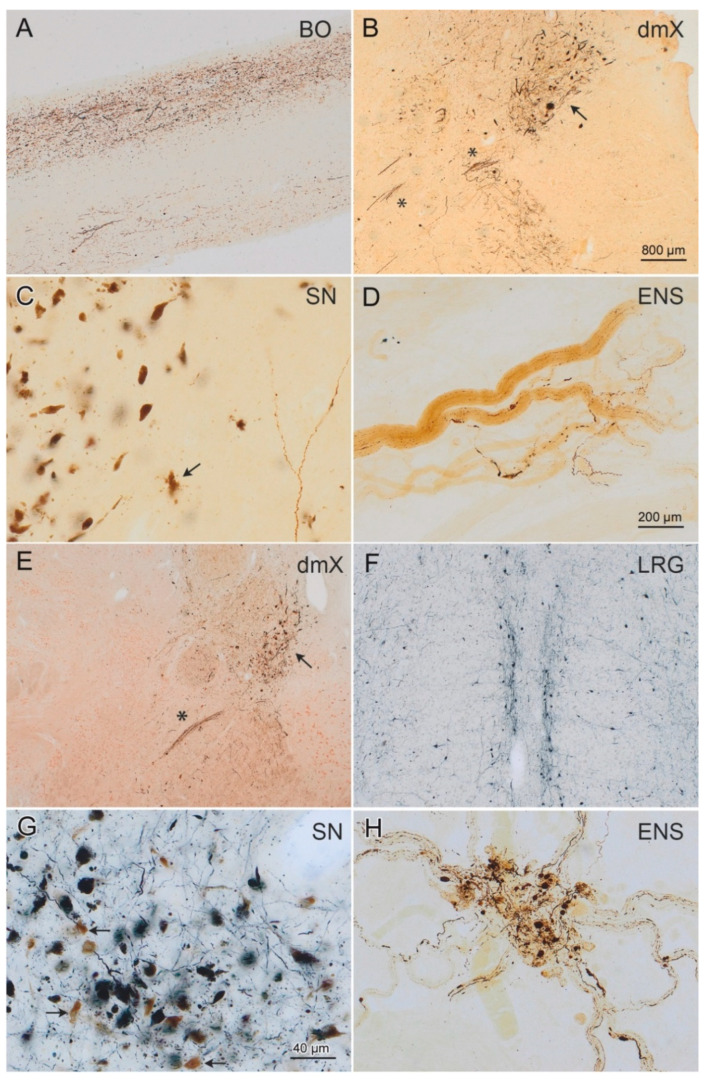
α-Synuclein-immunopositive Lewy neurites/bodies in *n* = 3 cases staged for LBD and tested for amplification. (**A**) Lewy pathology (DAB, brown chromogen) in the olfactory tract and anterior olfactory nucleus of case 5 (Table 1, ILBD stage 3, female, 63 years of age). These regions and all others analyzed, with the exception of the gastric Auerbach plexus (ENS), showed evidence of amplification (Figure 2). (**B**–**D**) Case 12 (Table 1, PD stage 3, male, 65 years of age) displayed Lewy pathology (DAB, brown chromogen) in the dorsal motor nucleus of the vagus nerve (dmX, arrow in (**B**) and also in axons of the vagus nerve (asterisk* in (**B**)). (**C**) Moderate pathology (at far lower right, branching Lewy neurites) with nascent neuronal loss (arrow points to extraneuronal neuromelanin granules, marking the site at which a dopaminergic nerve cell has been lost) was present in the pars compacta of the substantia nigra (SN). Amplification was not detected in the dmX, locus coeruleus, or SN of this individual (Figure 2). (**D**) By contrast, the Auerbach plexus of the stomach displayed Lewy neurites and amplification with a fibril-like profile (Figures 2 and 4). (**E**) Lewy pathology in the dmX (arrow in **E**; note also the immunopositive vagus nerve axons, asterisk*) of case 18 (Table 1, PD stage 5, female, 84 years of age). This individual displayed amplification in all regions analyzed (Figure 2) and, as in case 5, both ribbon-like and fibril-like profiles (Figures 5 and 6). (**F**–**H**) Case 11 (Table 1, PD stage 5, female, 68 years of age), in the great raphe nucleus (located at both sides of the midline in (**F**)), and in the pars compacta of the SN. (**G**) Amplification occurred with the presence of ribbon-like (dmX) and fibril-like profiles (great raphe nucleus) (Figures 2, 5, and 6). (**H**) Severe Lewy pathology in the gastric Auerbach plexus of a tangential section. Lewy pathology appears bluish-black (SK-4700, dark blue chromogen) in (**F**,**G**) and dark brown (chromogen DAB) in (**H**). The arrows in (**G**) point to intact dopaminergic neurons that contain neuromelanin. The scale bar in (**B**) applies to (**E**,**F**). The bar in (**D**) is also valid for (**A**,**H**); the scale bar in (**G**) applies to (**C**). 100 µm polyethylene glycol-embedded tissue sections (**A**–**C**,**E**,**F**), 100 µm cryosections (**D**,**H**). For data pertaining to seeding efficiency of specific tissues and lag phase, see Figure 3.

Histological slides were viewed and the inclusion body pathology and neuronal loss assessed semiquantitatively with an Olympus BX61 microscope (Olympus Optical, Tokyo, Japan) (Table 2). Digital micrographs of IHC-stained sections (Figure 1) were taken with an Olympus XC50 camera using the Cell D^®^ Imaging Software (Olympus, Münster, Germany). The extended focal imaging (EFI) function was used for stacks of images at different optical planes (Cell D Imaging Software, Olympus, Münster, Germany). The EFI algorithm extracts the image features with the sharpest contrast from all layers of the stack and merges them into a single image.

### 2.4. Punch Biopsies for Biochemical Analysis

From each neuropathologically staged case, including controls, 4 mm punch biopsies (Kai Industries Co, Ltd., Seki, Japan) were collected free-floating in aqua dest from unstained tissue sections adjacent to those used for IHC. The sections contained each of the six regions listed in Table 2. To avoid possible cross-contamination between individuals and anatomical regions, punch biopsy tools were utilized only once for each sample. Samples were encoded and all subsequent preparation and assays were performed in a blinded fashion. Tissue punches were stored in 1× TBS at 4 °C until use.

### 2.5. Brain Tissue Homogenization

Sections of fixed tissues were washed several times by centrifugation (15,000× *g* for 15 min) and resuspension in 150 mM KCl, 50 mM Tris-HCl, pH 7.5, to completely remove paraformaldehyde (PFA). After several washes, the sections were weighed in a Safelock 2 mL Biopur tube (Eppendorf, Montesson, France). The samples were diluted twenty-five times in a PMCA buffer (150 mM KCl, 50 mM Tris-HCl pH 7.5) in order to obtain a homogenate at 4% (weight:volume). The homogenization was performed by sonication using the SFX 150 Cell Disruptor sonicator with a 3.17 mm microtip probe (Emerson, Bron, France) for 10–60 s, with 1 s pulses followed by 1 s pauses in a biosafety level 3 environment (BSL-3). The homogenates were aliquoted and immediately frozen in liquid nitrogen before storage at −80 °C. All contaminated surfaces were cleaned with SDS (1%) [45].

### 2.6. Protein Misfolding Cyclic Amplification (PMCA) Assay

All operations were performed in BSL-3. Fixed tissue homogenates were diluted in PMCA buffer (150 mM KCl, 50 mM Tris-HCl, pH 7.5) containing monomeric αSyn (100 µM) to a final concentration of 2% (weight:volume), equivalent to 6 mg of fixed tissue, as described previously for unfixed tissues [31]. The sample was split in two tubes of PCR strips (BIOplastics, Landgraaf, The Netherlands). PMCA amplification was performed in duplicates for each patient using the Q700 generator and a 431MPX horn (Qsonica, Fisher Scientific, Illkirch, France). The power of the horn was set to 30% of maximal amplitude. The program of amplification consisted in 15 s of sonication and a 5-min pause at 31 °C. Every hour, 5 µL were withdrawn from each tube and diluted in 300 µL of 10 µM of Thioflavin T (ThT). The amplification was monitored by measuring ThT fluorescence using a Cary Eclipse Fluorescence Spectrophotometer (Agilent, Les Ulis, France) with fixed excitation and emission wavelength at 440 nm and 480 nm, respectively.

### 2.7. Proteolytic Digestion

De novo assembled αSyn fibrils and ribbons as well as patients’ PMCA-amplified αSyn assemblies in 150 mM KCl, 50 mM Tris- HCl, pH 7.5, were treated at 37 °C by proteinase K (3.8 µg/mL) (Roche). Aliquots were removed at different time intervals following the addition of the protease and transferred into Eppendorf tubes maintained at 90 °C and containing a sample buffer (50 mM Tris-HCl, pH 6.8, 4% SDS, 2% beta-mercaptoethanol, 12% glycerol and 0.01% bromophenol blue) to arrest the cleavage reaction immediately. After the incubation of each tube for 5 min at 90 °C, the samples were processed to monitor the time course of αSyn cleavage by PAGE (15%) after staining with Coomassie blue (Sigma).

### 2.8. Transmission Electron Microscopy (TEM)

The morphology of the de novo assembled and PMCA-amplified αSyn assemblies was assessed by TEM in a Jeol 1400 transmission electron microscope following the adsorption onto carbon-coated 200 mesh grids and negative staining with 1% uranyl acetate. The images were recorded using a Gatan Orius CCD camera (Gatan Inc., Pleasanton, CA, USA).

### 2.9. Aggregated αSyn Quantification in Patients’ Brain Homogenates

The Cisbio FRET assay (Cisbio, Codolet, France, cat. # 6FSYNPEG) was used to quantify phosphorylated αSyn in patients’ brain homogenates, following the manufacturer’s recommendations. Briefly, the brain homogenates were diluted to 2% (weight:volume) in a lysis buffer provided in the HTRF kit. 16 µL of each diluted brain homogenates were loaded into a 96 wells plate and mixed with 4 µL of the FRET donor and acceptor antibodies in the kit. The plate was sealed with a film (CmlAB, Esbjerg, Danemark, cat. # 13076-9P-500) and incubated for 20 h at 20 °C without shaking in a Thermomixer comfort (Eppendorf, Montesson, France). After incubation, time-resolved FRET was measured upon excitation at 337 nm using a plate reader (CLARIOstar, BMG Labtech, Ortenberg, Germany). The HTRF signal was recorded at two different wavelengths (665 nm and 620 nm). The amount of aggregated αSyn was derived from the 665/620 nm fluorescence ratio and multiplied by 10,000.

### 2.10. Frozen Brain Tissue Preparation

Frozen brain tissues were homogenized (10%, weight:volume) in 150 mM KCl, 50 mM Tris-HCl, pH 7.5, following the procedure described for fixed brain tissues after removal of PFA.

### 2.11. Preparation of Protein Assemblies

Protein Expression and Purification: The expression and purification of human wild-type αSyn was performed as previously described [46]. αSyn was incubated in 50 mM Tris-HCl, pH 7.5, 150 mM KCl, to obtain the fibrillar polymorph “fibrils”, or in 5 mM Tris-HCl, pH 7.5, for “ribbons”, at 37 °C, under continuous shaking in an Eppendorf Thermomixer set at 600 r.p.m for 4–7 days [19]. To generate fixed assemblies, fibrils and ribbons were diluted at 100 µM and fixed for 30 min at room temperature in 2% PFA. The fixation was stopped by adding Tris-HCl pH 7.5.

### 2.12. Statistical Analyses

All statistical analyses were performed using the Analysis Toolpak (Excel, Microsoft, Washington, DC, USA). Significance was declared at *p* < 0.05.

## 3. Results

A total of 29 autopsied patients were included in this study: 9 control patients (5 females and 4 males, 55.78 ± 7.19 years), 8 ILBD patients (2 females, 6 males, 60.25 ± 13.46 years), and 12 PD patients (7 females, 5 males, 72.08 ± 7.52 years). Age differed significantly between PD (72.08 ± 7.52 years) and controls (55.78 ± 7.19 years), whereas it did not differ significantly between ILBD (60.25 ± 13.46 years) and control (55.78 ± 7.19 years) patients. Whenever available, tissue samples were taken from the olfactory bulb (BO), including the anterior olfactory nucleus, the dorsal motor nucleus of the vagus nerve (dmX), the enteric nervous system (ENS, gastric cardia), the great raphe nucleus (lower raphe group, LRG), locus coeruleus (LC), and the substantia nigra, pars compacta (SN), from each individual.

### 3.1. HTRF Cisbio Assay

We first attempted to quantify pathological αSyn in the fixed brain tissues homogenates using the HTRF Cisbio assay [47]. No signal could be detected in contrast with equivalent frozen PD patient tissues (Figure S1).

### 3.2. PMCA Amplification

**Figure 2:**
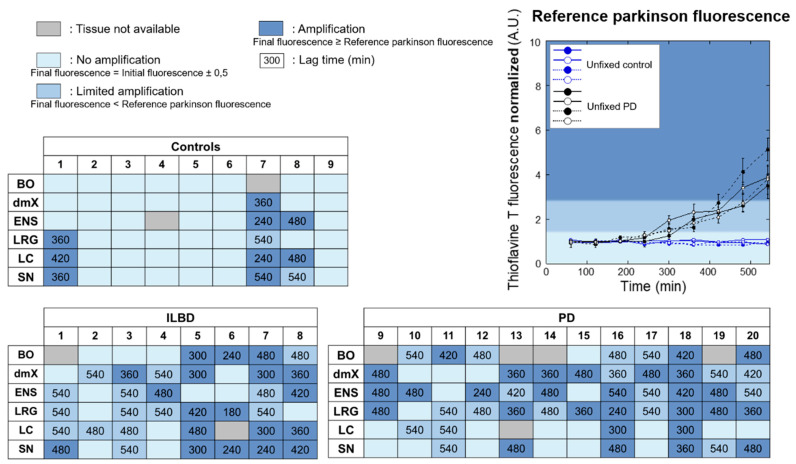
Graphical summary of the PMCA amplification reactions. PMCA was performed using biopsy homogenates from control, ILBD, and PD patients. The individual assembly kinetics from each tissue are presented in Figures S2–S4, where each curve represents an average of two replicates, ± SD. PMCA efficacy was considered high when the assembly kinetics lay within the dark blue surface area. PMCA efficacy was considered limited when the assembly kinetics lay within the lighter blue surface area. The surface area where no amplification was observed is shown in light blue. Representative amplification reactions from fresh brain homogenates from control and PD patients are shown. When tissue from a given region was unavailable, the corresponding box was labeled in gray. The duration of the lag time, in minutes, preceding the ThT fluorescence increase is provided in each box. No difference could be found between the PMI of CTL/ILBD/PD (*p* = 0.335). To analyze the effect of PMI on amplification, four groups (PMI < 20 h; 20 h < PMI < 30 h; 30 h < PMI < 40 h; and PMI > 40 h) were considered. Each group contains four or five ILBD/PD cases. No significant difference could be detected between these groups (*p* = 0.149). The *p*-value was calculated using the one-way analysis of variance (ANOVA) test.

The capacity of homogenized fixed tissues from different brain regions (2%, weight:volume) to seed the aggregation of monomeric αSyn was monitored by Thioflavin T (ThT) binding. The reaction efficacy was derived from the ThT fluorescence increase and the lag phase length preceding seeds elongation and amplification in the PMCA reaction. Amplification was considered efficient when the final ThT fluorescence signal was greater than, or equal to, the PD reference. Amplification was considered limited if the final ThT fluorescence signal was greater than controls but lower than the PD reference (Figure 2, Figures S2–S4).

**Figure 3:**
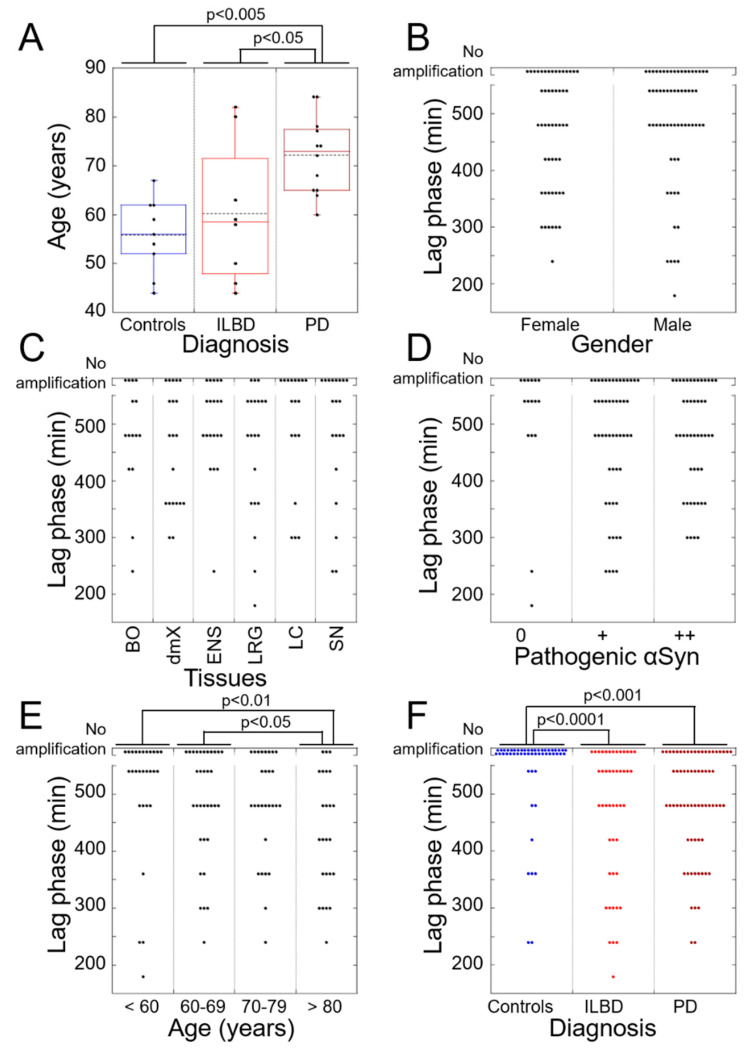
Statistical analysis of the data. (**A**) Age of patients according to diagnosis. No significant difference could be detected between the age of controls (CTL) and ILBD cases (CTL = 55.8 ± 7.2 years; ILBD = 60.3 ± 13.5 years; CTL vs. ILBD *p* = 0.365). PD patients were significantly older than controls or ILBD cases (PD = 72.1 ± 7.5 years; CTL vs. PD *p* = 0.0010; ILBD vs. PD *p* = 0.0153). (**B**) Seeding propensities of tissues according to sex. The lag phase for all females or males (ILBD, PD) was analyzed. No difference could be detected between females and males (476.5 ± 109.8 min and 491.6 ± 110.3 min; *p* = 0.472). (**C**) Seeding propensities according to region. The lag phase for all ILBD and PD patients’ tissues was analyzed. No difference was detectable between the regions (BO = 484 ± 103.8 min; dmX = 462 ± 107.5 min; ENS = 504 ± 85.7 min; LRG = 459 ± 118.9 min; LC = 506.7 ± 111.8 min; SN = 495 ± 119.8 min; *p* = 0.647). (**D**) Seeding propensities according to the load of pathogenic αSyn. The lag phase for all ILBD and PD patients’ tissues was analyzed. The pathogenic αSyn load was derived from Table 2 by semiquantitative assessment of LBs and LNs as follows: 0 = not detectable, + = mild to moderate, ++ = severe. No difference could be detected (“0” = 510 ± 121.9 min; “+” = 477.6 ± 114.6 min; “++” = 483.9 ± 99.6 min; *p* = 0.598). (**E**) Seeding propensities according to the age of ILBD and PD cases. The lag phase for all ILBD and PD patients’ tissues was analyzed. Four age groups were defined: ≤60 (*n* = 5), 60–69 (*n* = 6), 70–79 (*n* = 5) and ≥80 (*n* = 4) years of age. No difference could be detected between the groups ≤60, 60–69, 70–79 (“≤60” = 514.3 ± 116.1 min; “60–69” = 492.7 ± 105.2 min; “70–79” = 488.6 ± 99.8 min; “≤60” vs. “60–69” *p* = 0.447; “≤60” vs. “70–79” *p* = 0.350; “60–69” vs. “70–79” *p* = 0.832) or between the groups 70–79 and ≥80 (“≥80” = 435 ± 105.7 min; “70–79” vs. “≥80” *p* = 0.079). The average lag phase was significantly shorter for patients ≥80 than for patients ≤60 years of age or aged 60–69 years (“≤60” vs. “≥80” *p* = 0.0086; “60–69” vs. “≥80” *p* = 0.0436). (**F**) Seeding propensities according to patient diagnoses. The average lag phase was significantly shorter for ILBD or PD cases than for controls (CTL= 560.8 ± 90.3 min; ILBD = 473.5 ± 123.4 min; PD = 492.5 ± 99.7 min; CTL vs. ILBD *p* = 0.000065; CTL vs. PD *p* = 0.00058). No difference was found between ILBD and PD cases (ILBD vs. PD p = 0.345). One-way analysis of variance (ANOVA) test and Fisher’s LSD post hoc test were used for all statistical analyses.

Overall, ILBD patients were younger than their PD counterparts (Figure 3A). With regard to sex, there were no significant differences between ILBD, PD, and controls (Figure 3A). We did not observe statistically significant differences in the seeding propensities of tissues originating from males or females (Figure 3B). No statistically significant difference in the seeding efficiency of specific tissues that we analyzed was observed (Figure 3C). Furthermore, we did not observe a relationship between the load of pathogenic αSyn deposits and seeding efficiency (Figure 3D).

Four patients’ age groups were defined (≤60, 60–69, 70–79, and ≥80 years of age) (Figure 3E). The lag phase preceding assembly for patients ≥80 was significantly shorter than that for patients ≤60 years old or aged 60–69, thereby suggesting the presence of increased numbers of seeds or the higher seeding potential of pathogenic αSyn (Figure 3E).

Unexpectedly, and in contrast with what we observed in both of the frozen control samples used in this study as well as in samples (*n* = 5) from another study [31], a high seeding efficacy was observed in three of nine fixed control samples. Limited to high seeding was observed in at least two fixed tissues from all ILBD and PD patients (Figure 2), in agreement with what we observed in the frozen PD samples used in this study (*n* = 2) and from a further prior study (*n* = 14) [31].

When averaged, the lag phase for control cases was significantly longer than that of ILBD or PD cases (Figure 3F). When this statistical analysis was limited to the control cases where we observed amplification, no statistically significant differences were observed. This may suggest that, although asymptomatic, the controls do not differ from ILBD patients.

### 3.3. PMCA-Amplified Assemblies Characterization by TEM

**Figure 4:**
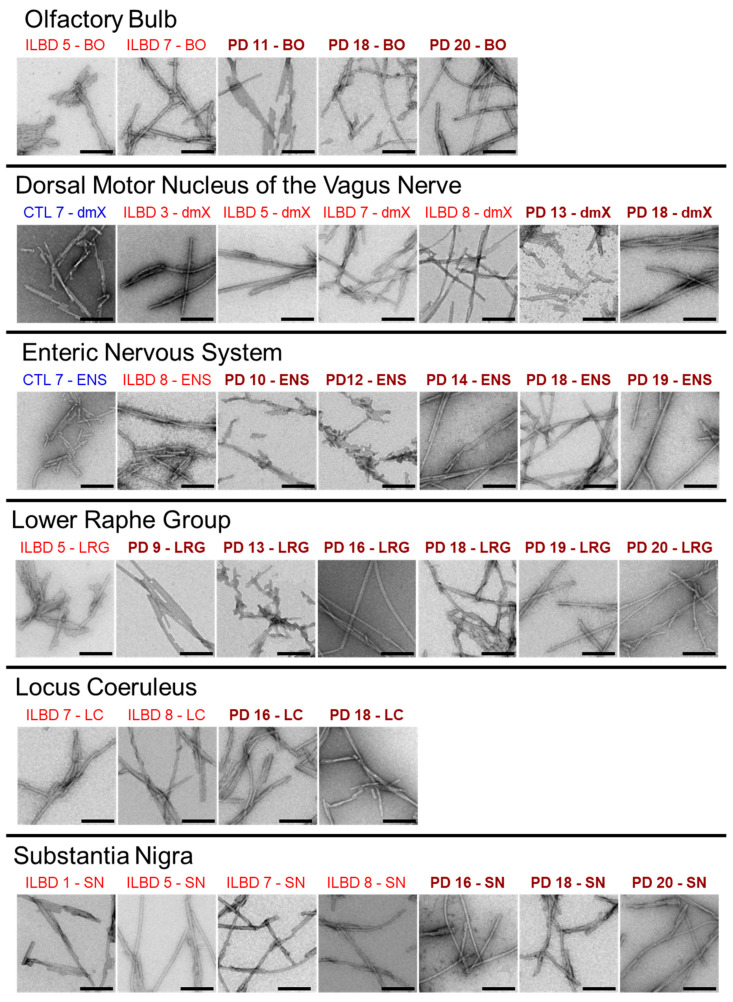
Characterization of PMCA-amplified αSyn assemblies by transmission electron microscopy. Uranyl acetate negatively stained electron micrographs of αSyn assemblies obtained by PMCA amplification in different regions from ILBD and PD patients. Attention was directed to the fibril aspect in TEM, e.g., twisted, flat or not flat, whereas the polymorph fibrils are not twisted or flat in contrast to the polymorph ribbons. Scale bar = 200 nm.

We next imaged the resulting fibrillar αSyn assemblies by means of TEM after negative staining. The TEM analysis revealed the presence of 6–8 nm wide fibrillar assemblies that often stacked laterally into bundles (Figure 4). In some instances, twists and/or a groove running along the length of the fibril could be distinguished (Figure 4), thereby suggesting that the fibrillar assembly is flat. The EM analysis did not reveal PD or ILBD-specific differences in the shape of the fibrils. The latter contrasts with the disease-specific differences in αSyn fibril morphology we previously reported upon PMCA amplification of frozen PD and DLB patients’ brains [31].

### 3.4. PMCA-Amplified Assemblies and Proteolytic Profiling

**Figure 5:**
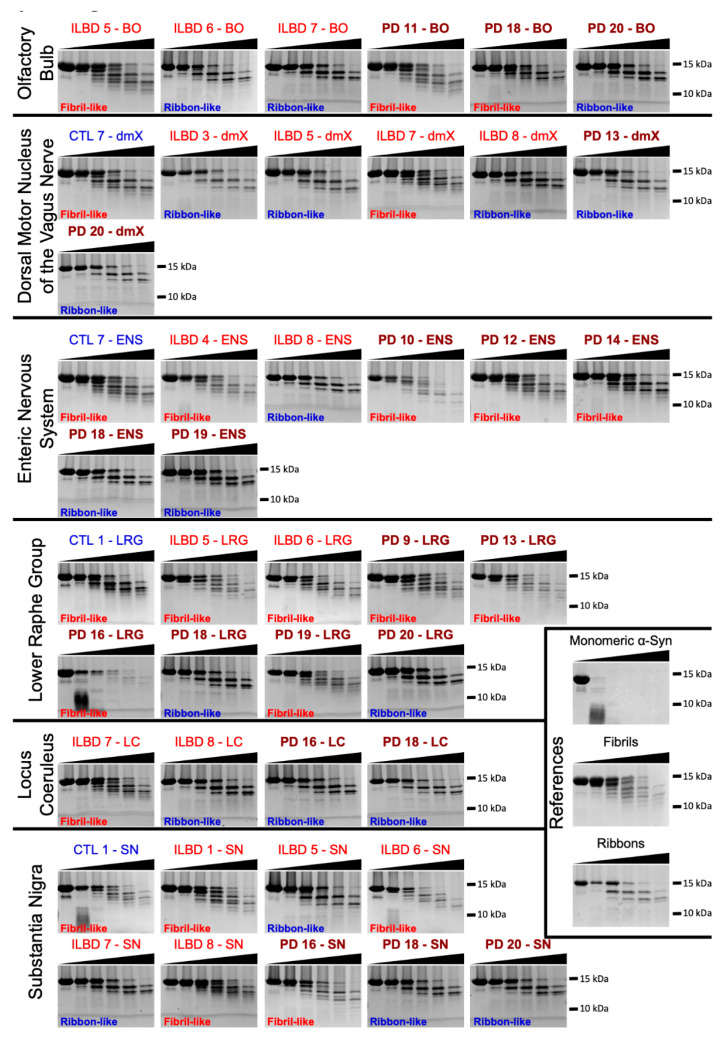
Characterization of PMCA-amplified αSyn assemblies by proteolytic profiling. Limited proteolytic patterns of αSyn assemblies obtained by PMCA amplification from different tissues of ILBD and PD patients, from recombinant monomeric and de novo generated αSyn fibrils and ribbons. Monomeric αSyn concentration is 100 μM, and proteinase K concentration is 3.8 μg/mL in all reactions. Samples were withdrawn from the reaction immediately after PK addition (leftmost lane) and at 1, 5, 15, 30 and 60 min, from left to right, in all panels. A SDS-PAGE analysis was performed as described in the “Materials and methods” section, and the gels were stained with Coomassie blue. The molecular weight markers are shown on the left.

**Figure 6:**
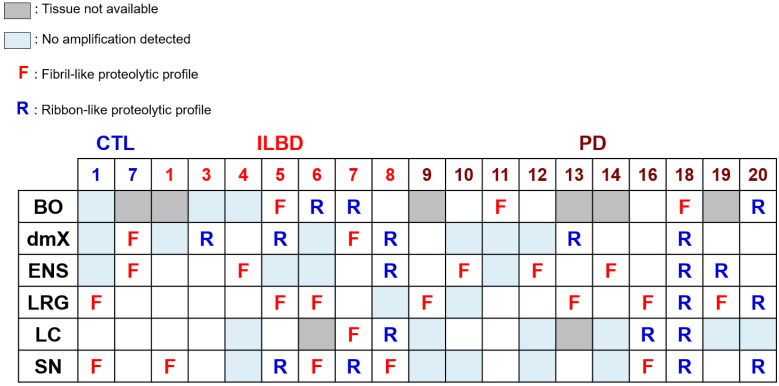
Graphical summary of the proteolytic profiling of αSyn assemblies amplified by PMCA. The limited proteolytic patterns of αSyn assemblies obtained by PMCA amplification from different tissues of ILBD and PD patients were compared to those of de novo assembled αSyn fibrils and ribbons. When the pattern resembled that of fibrils, the box was labeled F (“fibril-like”). When the pattern resembled that of ribbons, the box was labeled R (“ribbon-like”). When a region displayed no propensity for seeding in PMCA reactions, the corresponding box was labeled in light blue. When tissue from a given region was unavailable, the corresponding box was labeled in gray. White (blank) boxes indicate regions displaying amplification or limited amplification for which tissue was not subjected to TEM and limited proteolytic profiling.

We also fingerprinted the PMCA-amplified fibrillar αSyn assemblies derived from patients’ fixed brain tissue homogenates by limited proteinase K degradation. All limited proteinase K degradation patterns obtained can be said to resemble those of the reference fibrillar polymorphs assembled de novo from pure αSyn, which we have termed “fibrils” and “ribbons” elsewhere [19]. Thus, they were labeled “fibril-like” or “ribbon-like” (Figure 5). However, the limited proteolysis patterns of PMCA-amplified fibrillar αSyn assemblies from PD fixed brain tissue homogenates exhibited *both* fibril- and ribbon-like patterns, as did their ILBD counterparts (Figure 5). This contrasts with the strain-specific fingerprints (patterns) we reported for fibrillar assemblies derived from the PMCA amplification of frozen PD and DLB brain homogenates [31]. In addition, in most cases, e.g., ILBD cases 5–8, but also PD cases 13, 16, 18, and 19 (Figure 6), either fibril- or ribbon-like patterns were seen in different regions.

**Figure 7:**
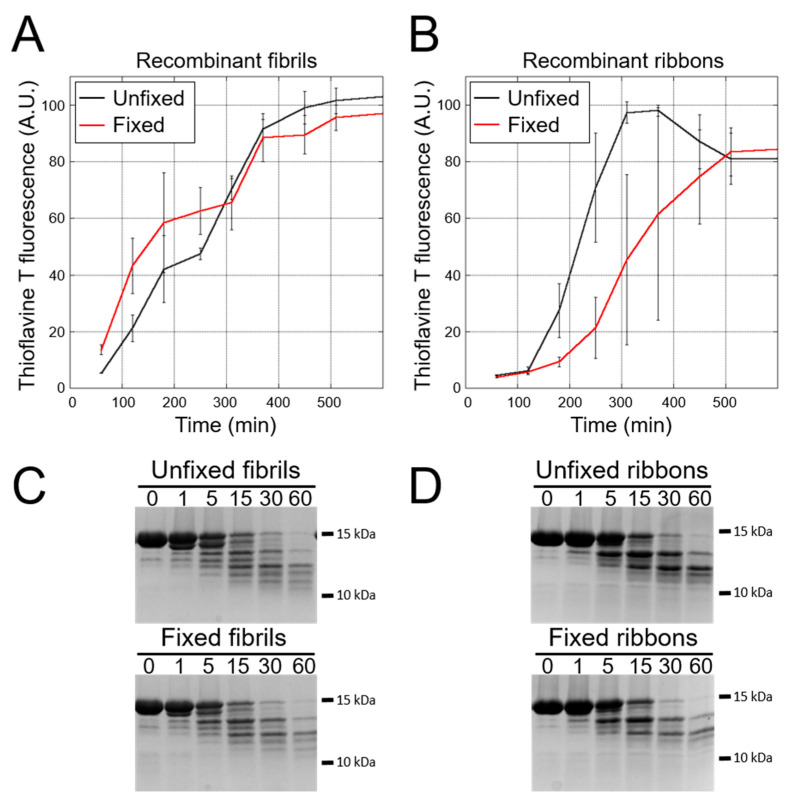
Seeding propensity of αSyn fibrils and ribbons assembled de novo before and after formalin treatment. The fibrillar polymorphs fibrils (**A**) and ribbons (**B**) were exposed to formalin as described in the “Material and methods” section, and their elongation propensity was assessed by Thioflavin T binding under the PMCA conditions. Each curve represents an average of two replicates, ±SD. Limited proteolytic patterns of αSyn fibrils and ribbons obtained by PMCA amplification from fresh (upper panel) or formalin-treated (lower panel) αSyn fibrils (**C**) and ribbons (**D**). Monomeric αSyn concentration is 100 μM, and proteinase K concentration is 3.8 μg/mL in all reactions. Samples were withdrawn from the reaction at the time indicated in min. A SDS-PAGE analysis was performed as described in the “Materials and methods” section, and the gels were stained with Coomassie blue.

Strain variance within one and the same individual may be the consequence of the compromised templating capacity of the seeds following cross-linking. We therefore compared the seeding propensity of αSyn polymorphs fibrils and ribbons assembled de novo before and after fixation and the proteolytic patterns of the resulting fibrillar assemblies (Figure 7). Our results show unequivocally that fixation affects neither the seeding potential of the different αSyn fibrillar polymorphs, as assessed by ThT binding, nor the true templating of their intrinsic structure, as assessed by limited proteolytic fingerprinting (Figure 7).

## 4. Discussion

Here, we could show that αSyn deposits retain their seeding potential after the formalin fixation of tissues. This is in agreement with previous reports, in which transgenic mice developed synucleinopathies upon injection of formalin-fixed brain tissue homogenates from mice spontaneously developing αSyn-rich deposits [48]. Unlike the results we recently reported using frozen brain tissues from control cases [31], three of the nine fixed control samples here exhibited a significant seeding propensity. Given that monomeric αSyn fails to assemble on its own or in the presence of frozen control brain homogenates under our experimental conditions, this propensity for seeding must be attributable to the fixation reaction. Indeed, the cross-linking agent may either induce the formation of αSyn oligomeric species with seeding potential, or it may potentiate pathogenic αSyn species activity. Cross-linking agents have indeed been demonstrated to stabilize pathogenic PrP and αSyn infectious particles [49,50,51,52].

The seeding propensities we quantified could not be correlated to the load of pathogenic αSyn, specific brain regions, or gender (Figure 3). The lag phase preceding the assembly for patients ≥80 of age was significantly shorter than that for younger patients. This suggests that either the number of pathogenic seeds increases in the brains of such patients or that the seeds acquire a higher seeding propensity with time. Seeding activity did not diminish in late-stage disease (Figure 2). In three control cases where seeding was observed, the length of the lag phase preceding the assembly was statistically indistinguishable from that of ILBD or PD cases. No seeding was observed in the vast majority of fixed (this study) or fresh [31] tissues from control patients. We previously reported seeding in a biopsy from the antrum of a patient collected 10 years before the patient exhibited PD symptoms [32]. Thus, the three seeding-positive control patients might still have been asymptomatic at autopsy and the level of Lewy pathology in the tissues collected below the threshold for immunohistochemical detection. Alternatively, PFA fixation might stabilize an otherwise labile seed.

PMCA-amplified fibrillar αSyn assemblies from fixed ILBD and PD patient tissues did not exhibit distinct shapes that could be defined as being characteristic of each group. This suggests that while the seeding potential of fixed brain tissues is preserved, the intrinsic structure of the seeds fades upon cross-linking, yielding similar fibrillar assemblies. Albeit indistinguishable based on their morphology, the limited proteolytic patterns of the fibrillar αSyn assemblies derived from different ILBD and PD brain regions varied significantly. This, in turn, points to the existence of distinct strains within different central or peripheral nervous system regions of some individuals (Figure 6), given that the true templating of the intrinsic structure of de novo assembled fibrils and ribbons is not impacted by fixation (Figure 7).

The prion protein PrP forms a heterogeneous ensemble of high molecular weight species in human prion diseases [53]. It has been shown that the host conditions and the environment define strain selection, amplification, and maintenance [54]. The finding that the strains vary from region to region within the same individual may indicate that spreading from one region to another along axonal connectivities alone does not account for pathology progression. For example, the dmX displayed in one and the same individual (see ILBD case 5 and PD case 13 in Figure 6) a ribbon-like profile, whereas the LRG displayed a fibril-like profile. αSyn aggregation is stochastic. Several aggregation events may occur in different tissues within any given time frame, thus leading to the observation we report. The exactitude with which different strains template αSyn aggregation may also depend on the regional and/or cellular context; in particular, αSyn expression levels [55] may represent another explanation for our findings.

The present study has potential limitations. The risk of inexactly templating the aggregation of αSyn resulted in our decision not to further systematically characterize the assemblies derived from reactions where amplification was limited, i.e., 34 samples out of a total of 165, 74 of which showed no amplification (Figure 2). In addition, our observation of “ribbon-like” and “fibril-like” assemblies in 8 of 19 individuals led us to conclude that the possible benefits to be gained by performing further amplification, TEM, and limited proteolytic profiling on every region from all cases where tissue was available and amplification was present (37 of 80 samples in Figure 6) did not justify the considerable resources required.

Finally, and notably, the SN did not display a higher amplification/seeding predilection in some ILBD cases than the ENS, LRG, or BO (cases 1, 5, 7 in Figure 2), thereby suggesting that the SN may not be as selectively vulnerable in ILBD as other regions in early disease. By contrast, at later PD stages, SN neurons may either be lost before the accumulation of seeds can take place—this could account for the lower seeding rates seen there in some PD cases in comparison to the seeding activity seen in other regions (cases 16, 18, and 20 in Figure 2)—or, alternatively, exogenous seeds from dying SN nerve cells may somehow be taken up by other still vital SN neurons that have a propensity for seeding (PD cases 13 and 19 in Figure 2). Apart from selective vulnerability, it is also conceivable that a given region could demonstrate a stronger αSyn seeding predilection than another while possessing some form of cellular “resistance” to the effects of seeding.

## Figures and Tables

**Table 1:** Demographic and clinico-neuropathological data from the *n* = 29 cases studied.

	Case	f/m	Age	PMI	Brain wt	NFT	Aβ	α-Syn	Diagnoses
**Control**	1	♀	44	18	1365	0	0	0	malignant neoplasm
	2	♂	46	na	1480	0	0	0	sepsis (abscess)
	3	♂	52	41	1332	I	0	0	malignant neoplasm
	4	♀	54	22	na	I	0	0	malignant neoplasm
	5	♂	56	18	1352	I	0	0	coronary artery disease
	6	♀	59	20	1401	I	0	0	sepsis, cardiac failure
	7	♀	62	32	1364	I	0	0	malignant neoplasm
	8	♂	62	7	1410	I	0	0	cardiac disease
	9	♀	67	4	1180	I	0	0	acute myeloid leukemia
**Lewy Body Disease**	1	♂	46	71	1500	0	0	2	ILBD, pericardial tamponade
	2	♂	50	6	1250	0	0	2	ILBD
	3	♂	58	39	1438	I	0	2	ILBD, myocardial infarction
	4	♀	59	24	1100	0	0	1	ILBD, malignant lymphoma
	5	♀	63	28	1150	I	0	3	ILBD
	6	♂	44	26	1400	II	0	1	ILBD, aspiration pneumonia
	7	♂	82	16	1192	III	1	3	ILBD, malignant neoplasm
	8	♂	80	na	1400	III	0	3	ILBD, coronary artery disease
	9	♂	60	32	1470	I	0	5	PD, bronchopneumonia
	10	♀	64	96	1318	II	1	4	PD, acute myeloid leukemia
	11	♀	65	8	1175	II	2	4	PD, pulmonary embolism
	12	♂	65	34	1500	I	0	3	PD, myocardial infarction
	13	♀	68	10	1145	III	3	5	PD, myocardial infarction
	14	♀	72	12	1214	I	2	5	PD, bronchopneumonia
	15	♀	74	42	1294	II	2	5	PD, AGD, myocardial infarction
	16	♀	78	21	1130	III	1	3	PD, pneumonia
	17	♂	84	28	1200	II	2	4	PD, aspiration pneumonia
	18	♀	84	30	1045	V	3	5	PD, AD, AGD
	19	♂	77	na	1232	II	0	5	PD
	20	♂	74	93	1344	V	4	5	PD, AD, absolute arrhythmia

The study cohort (*n* = 29) included two groups: 9 controls (5 females, 4 males, age range 44–67 years) and 20 cases with Lewy body disease (LBD), including 8 with incidental Lewy body disease, ILBD (2 females, 6 males, age range 44–82 years), and 12 with sporadic Parkinson’s disease, PD (7 females, 5 males, age range 60–84 years). Abbreviations: f/m—female/male; Age—age in years; PMI—Post-mortem interval in hours; Brain wt—fresh brain weight in grams; NFT—Alzheimer’s disease-related neurofibrillary tangle stage 0-VI using a modified Gallyas silver-iodide staining [36,37]; Aβ—amyloid-β deposition phase 0–5 using 4G8 immunohistochemistry (IHC) [38]; α-syn—Parkinson’s disease-related neuropathological stage 0–6 using α-synuclein IHC [2,39]; AD—Alzheimer’s disease; AGD—argyrophilic grain disease; na—not available.

**Table 2:** Semiquantitative assessment of Lewy pathology in *n* = 20 cases with Lewy body disease.

Case	f/m	Age	NFT	Aβ	PD	Diagnoses	ENS	BO	dmX	LRG	LC	SN
1	♂	46	0	0	2	ILBD	0	++	+	+	+	0
2	♂	50	0	0	2	ILBD	0	++	+	+	+	0
3	♂	58	I	0	2	ILBD	0	+	+	+	+	0
4	♀	59	0	0	1	ILBD	0	+	0	0	0	0
5	♀	63	I	0	3	ILBD	+	++	+	+	+	+
6	♂	44	II	0	1	ILBD	0	+	0	0	na	0
7	♂	82	III	1	3	ILBD	+	+	++ *	++	+	+
8	♂	80	III	0	3	ILBD	+	++	++ *	+	+	++
9	♂	60	I	0	5	PD	++	++	++ *	+	[++]	[++]
10	♀	64	II	1	4	PD	0	+	+	++	[++]	+
11	♀	65	II	2	4	PD	+	++	+	+	[+]	[++]
12	♂	65	I	0	3	PD	+	++	++ *	++	++	+
13	♀	68	III	3	5	PD	++	++	++ *	++	[++]	[++]
14	♀	72	I	2	5	PD	++	++	++ *	+	[+]	[++]
15	♀	74	II	2	5	PD, AGD	+	++	++ *	++	[++]	[++]
16	♀	78	III	1	3	PD	+	++	+	+	+	[+]
17	♂	84	II	2	4	PD	+	++	++ *	++	[++]	[++]
18	♀	84	V	3	5	PD, AD, AGD	+	++	++ *	++	[++]	[++]
19	♂	77	II	0	5	PD	+	++	++ *	++	[++]	[++]
20	♂	74	V	4	5	PD, AD	+	+	+	+	[+]	[+]

Lewy pathology in neuronal somata (Lewy bodies, LBs) and in nerve cell processes (Lewy neurites, LNs) as well as neuronal loss were semiquantitatively assessed in 100 µm immunostained tissue sections as follows: none or not detectable 0, mild to moderate +, severe ++. Neuropathological staging is based on the regional distribution (i.e., extent) of pathology [44]. Abbreviations: f/m—female/male; Age—age in years; NFT—Alzheimer’s disease-related neurofibrillary tangle stage 0-VI using a modified Gallyas silver-iodide staining [36,37]; Aβ—amyloid-β deposition phase 0–5 using 4G8 immunohistochemistry (IHC) [38]; α-syn—Parkinson’s disease-related neuropathological stage 0–6 using α-synuclein IHC [2,39]; diag—diagnoses; AD—Alzheimer’s disease; AGD—argyrophilic grain disease; ENS—gastrointestinal nervous system (gastric cardia); BO—olfactory bulb, including the anterior olfactory nucleus; dmX—dorsal motor nucleus of the vagal nerve; LRG—lower raphe group (great raphe nucleus); LC—locus coeruleus; SN—substantia nigra, pars compacta; na—not available. Brackets [] indicate neuronal loss. Asterisk* indicates the presence of syn-1 IHC-positive axons in the vagus nerve (N. X) of the dorsal motor nucleus (see Figure 1A,E, asterisks*).

## Data Availability

The data presented in this study are available herein and in the supplementary Figures S1–S4 (https://www.mdpi.com/xxx/s1).

## Supplementary Materials

The following are available online at https://www.mdpi.com/2073-4409/10/1/139/s1, Figure S1: Quantification of phosphorylated αSyn; Figure S2: Amplification of aggregated αSyn from fixed tissues by PMCA (olfactory bulb and dorsal motor nucleus of the vagus nerve); Figure S3: Amplification of aggregated αSyn from fixed tissues by PMCA (enteric nervous system and lower raphe nuclei); Figure S4: Amplification of aggregated αSyn from fixed tissues by PMCA (locus coeruleus and substantia nigra, pars compacta).
