# Supplementary material for: Seeding Propensity and Characteristics of Pathogenic αSyn Assemblies in Formalin-Fixed Human Tissue from the Enteric Nervous System, Olfactory Bulb, and Brainstem in Cases Staged for Parkinson’s Disease
Source: Cells. 2021 Jan 12;10(1):139. doi: 10.3390/cells10010139 (PMC7828121; doi:10.3390/cells10010139)
Supplement: Supplementary file 1 [file cells-10-00139-s001.pdf]

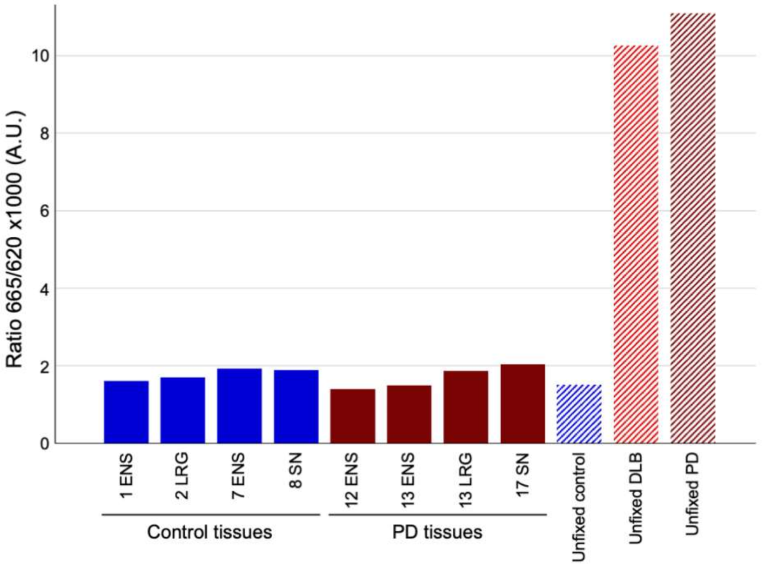

Figure S1. Quantification of phosphorylated αSyn.

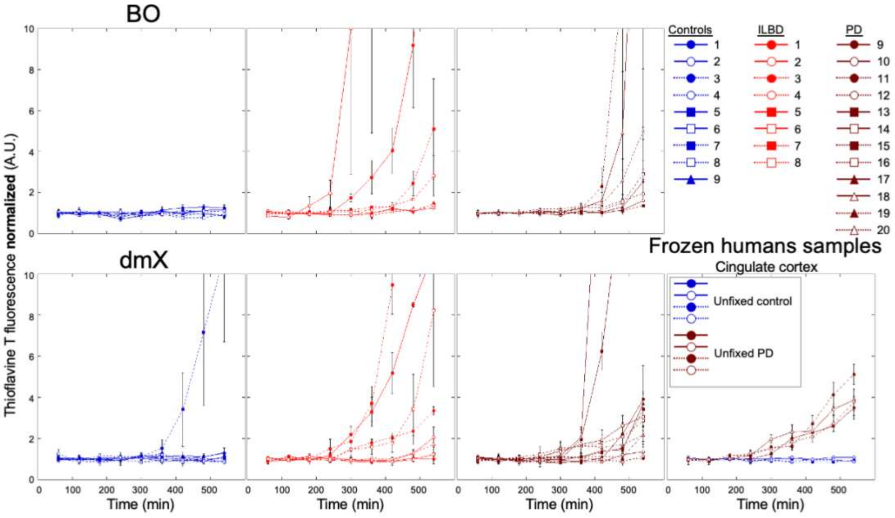

Figure S2. Amplification of aggregated αSyn from fixed tissues by PMCA (olfactory bulb and dorsal motor nucleus of the vagus nerve).

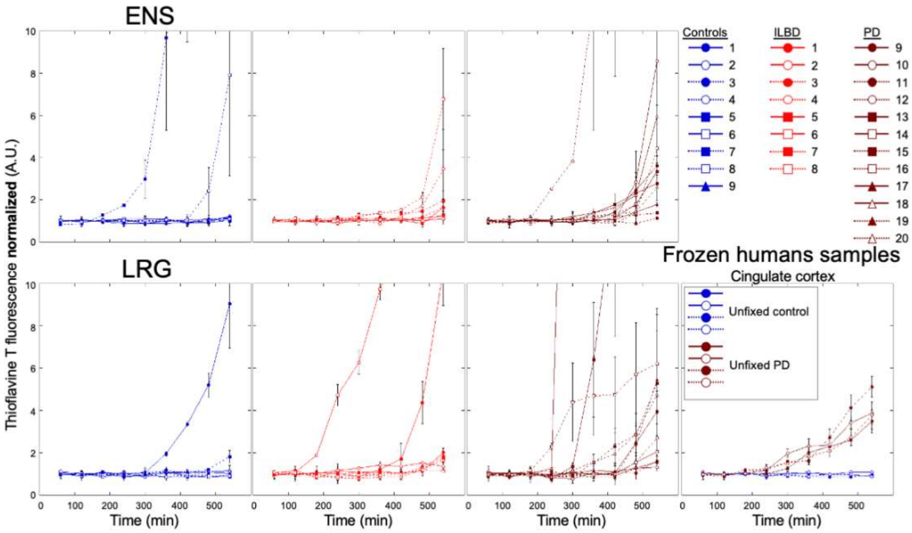

Figure S3: Amplification of aggregated αSyn from fixed tissues by PMCA (enteric nervous system and lower raphe nuclei)

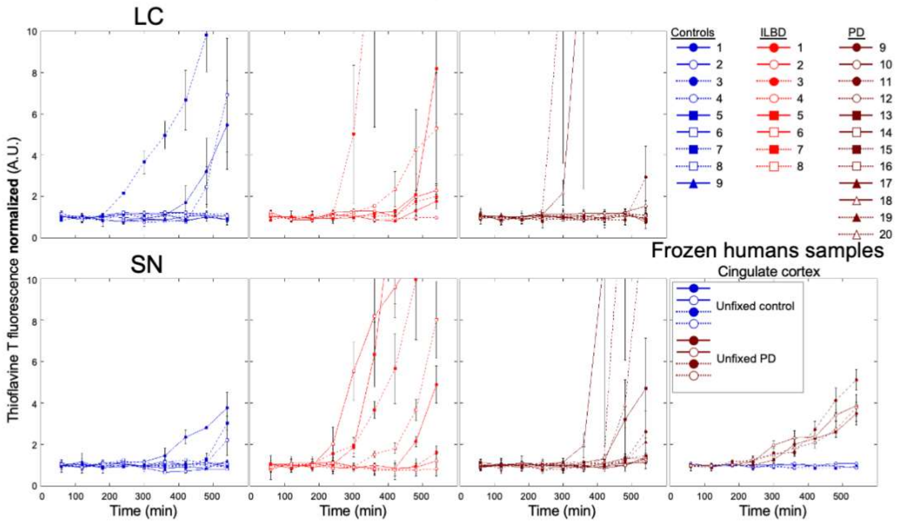

Figure S4: Amplification of aggregated αSyn from fixed tissues by PMCA (locus coeruleus and substantia nigra, pars compacta).
